# Breast Imaging Findings in Dermatofibrosarcoma Protuberans: A Case Report and Review of Literature

**DOI:** 10.7759/cureus.30175

**Published:** 2022-10-11

**Authors:** Elizabeth Pernicone MD, Kelly Fabrega-Foster MD

**Affiliations:** 1 Surgery, University of South Florida, Orlando, USA; 2 Breast Imaging, Tampa General Hospital, Tampa, USA

**Keywords:** skin malignancy, soft tissue sarcoma, dermatofibrosarcoma protuberans, dermatology, breast imaging

## Abstract

Dermatofibrosarcoma protuberans (DFSP) is a rare, indolent, cutaneous sarcoma originating in the dermis, and although nearly half of cases occur on the trunk, DFSP of the breast is exceedingly rare, and imaging findings may resemble primary breast neoplasms. In this case report, a previously healthy, middle-aged female patient presented to the clinic with the complaint of abnormal growth in her left breast, which had been gradually increasing in size over the course of four years. Imaging of the left breast demonstrated a large, exophytic, partially intradermal mass with internal vascularity, raising concern for a primary breast neoplasm. Ultrasound-guided core needle biopsy revealed a diagnosis of DFSP. She underwent successful left skin- and nipple-sparing mastectomy with complete resection of the mass with negative margins confirmed on surgical pathology.

Recognizing key features of DFSP on conventional breast imaging modalities, such as mammography and ultrasound, can be helpful in differentiating DFSP from primary breast neoplasms, but imaging findings alone may be nonspecific and biopsy is necessary for a definitive diagnosis. On mammography, DFSP typically presents as an exophytic, gently lobulated, non-calcified, and circumscribed mass. On sonographic examination, DFSP appears as a circumscribed, parallel-oriented mass that is hypoechoic relative to the surrounding fat, with intervening echogenic bands, posterior acoustic enhancement, and intralesional hypervascularity visualized on color Doppler. Although DFSP is slow-growing with a low incidence of metastatic disease, it has a high local recurrence rate and aggressive local resection is necessary to minimize the chance of recurrence.

## Introduction

Dermatofibrosarcoma protuberans (DFSP) is a rare, indolent, low- to intermediate-grade cutaneous sarcoma originating in the dermis, with an incidence rate of approximately four cases per million person-years [1]. Although nearly half of cases occur on the trunk, DFSP of the breast is exceedingly rare and its imaging appearance can be non-specific, making diagnosis by imaging alone difficult for clinicians [2,3]. In this case report and literature review, we detail breast imaging findings in a middle-aged woman who presented with a four-year history of a slowly enlarging, lobulated, and partially ulcerating mass of the left breast, which was proven on biopsy to be DFSP.

## Case presentation

**Figure 1:**
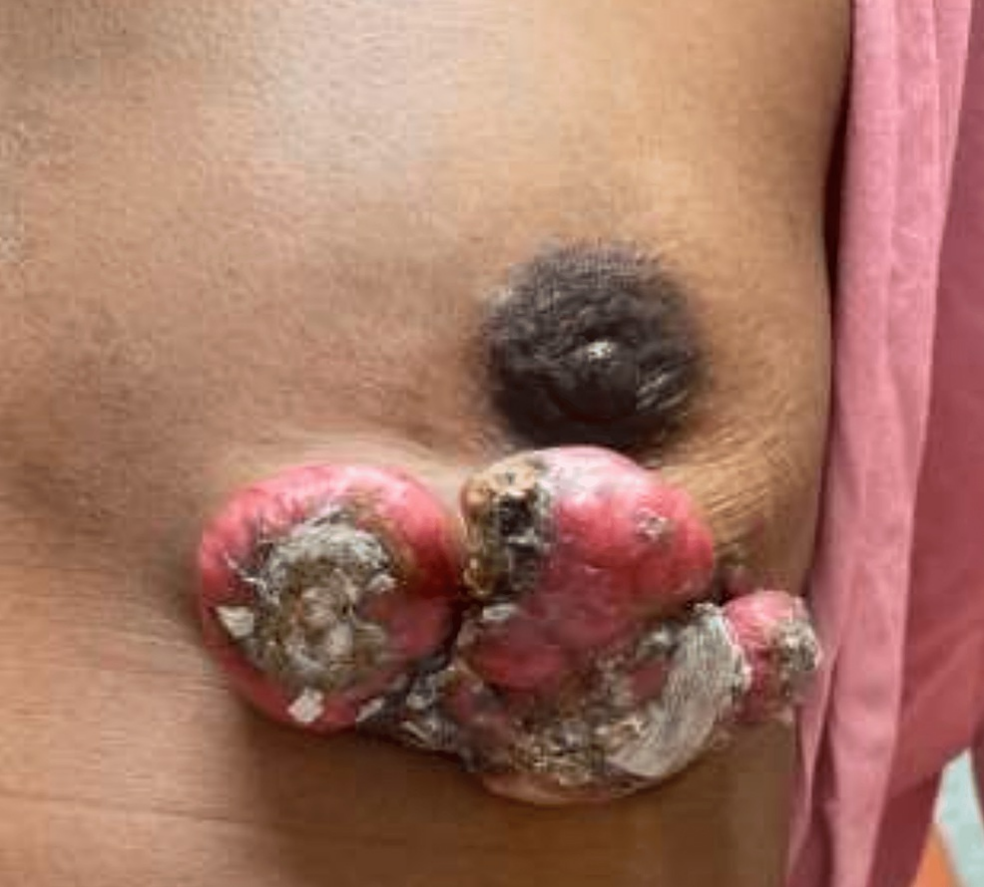
Physical Examination Findings at the Time of Initial Presentation in the Breast Clinic Findings on physical examination at initial presentation in the breast clinic. An erythematous, exophytic, ulcerated single large mass consisting of multiple sizeable, coalescing, pearly pink nodules with central ulceration is observed in the inferior left breast. The mass was tender to palpation, and the patient complained of intermittent bleeding from the lesion.

A previously healthy, middle-aged woman presented to her primary care physician with a complaint of a four-year history of abnormal growth in her left breast, which had been gradually increasing in size and was now tender to palpation. Initially, she reported the affected area had appeared as a small, erythematous plaque, for which she had been prescribed topical steroids with minimal improvement. The lesion continued to increase in size and, at the time of presentation to our breast clinic, there was a single large mass consisting of multiple sizeable, coalescing, pearly pink nodules with central ulceration (Figure 1).

**Figure 2:**
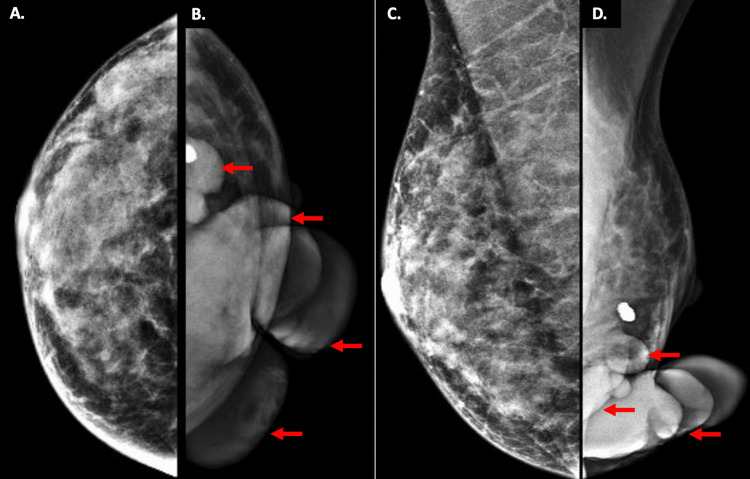
Bilateral Mammographic Examination Findings Images 2A (craniocaudal view) and 2C (mediolateral oblique view) show unremarkable findings in the patient’s right breast, which appears heterogeneously dense. Images 2B (craniocaudal view) and 2D (mediolateral oblique view) show part of a large, exophytic, lobulated mass (red arrows), which measured 6.5 cm in greatest diameter, located in the inferior part of the left breast. Complete visualization of the breast on mammography and compression views was not possible due to ulceration of the mass and the risk of bleeding.

As part of her breast imaging workup, full-field diagnostic bilateral mammography and left breast and axillary ultrasound were performed. The right breast was unremarkable (Figures 2A, 2C). In the left breast's lower inner quadrant, there was a large, exophytic, lobulated mass with circumscribed margins that could not be fully included in the field of view due to its size. On mammography, the included portions measured approximately 6.5 cm in diameter (Figures 2B, 2D).

**Figure 3:**
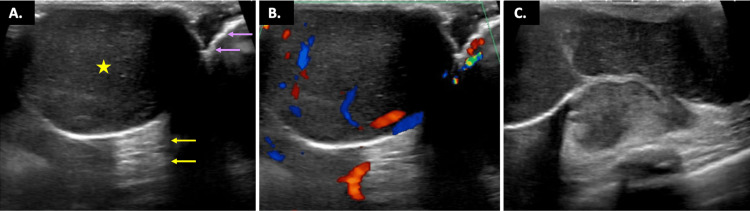
Sonographic Examination Findings in the Left Breast Image 3A, taken at 7:00, 5 cm from the nipple in the left breast, demonstrates a large, oval, circumscribed, parallel-oriented, hypoechoic mass (yellow star) with acoustic enhancement (yellow arrows) noted behind it. The dermal layer (lavender arrows) can also be seen cradling the mass, as it appears to invade from the dermis into the deeper subcutaneous tissues. Image 3B shows increased vascularity within the peripheral regions of the mass, in the same location in the left breast. Image 3C reveals a different view at 5:00, 5 cm from the nipple, depicting the lobulated appearance of this patient’s advanced DFSP left breast lesion.

Subsequent ultrasound revealed a solid, multilobulated mass with circumscribed margins and prominent posterior acoustic enhancement, predominantly situated in the dermis of the left breast between 5:00 and 8:00 (Figure 3). There was no associated lymphadenopathy.

**Figure 4:**
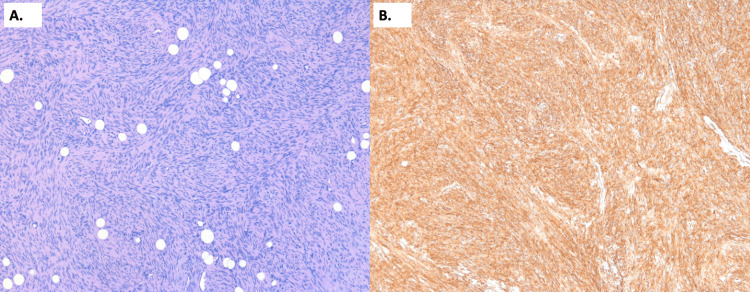
Histological Appearance of the Left Breast Lesion Image 4A demonstrates H&E staining of the patient’s left breast mass. A low-grade spindle cell neoplasm is seen involving the dermis and infiltrating between fat cells, without an epithelial component. Spindle cells are densely arranged in a storiform arrangement. Image 4B demonstrates positive CD34 immunohistochemical staining of the spindle cells, as is classically described in cases of DFSP. DFSP: dermatofibrosarcoma protuberans

She underwent an ultrasound-guided core needle biopsy of the mass, which confirmed a diagnosis of dermatofibrosarcoma protuberans. She was promptly referred to breast surgery for management and underwent a skin- and nipple-sparing left mastectomy with sentinel node excision. Surgical pathology revealed a 10.8 cm dermatofibrosarcoma protuberans, which appeared macroscopically confined to the skin (no grossly visible invasion of the underlying breast parenchyma) with negative resection margins and a negative sentinel node. Microscopic evaluation revealed a low-grade spindle cell neoplasm with mild cytologic atypia, involving the dermis and invading the subcutaneous tissue (Figure 4A). Immunohistochemical staining was positive for CD34, consistent with a diagnosis of DFSP (Figure 4B).

Following a successful left mastectomy, the patient was referred to medical oncology for continued management on an as-needed basis.

## Discussion

DFSP is a rare, indolent, low- to intermediate-grade, malignant, mesenchymal tumor originating in dermal fibroblasts [1]. It was originally described by Darier and Ferrand-Drakein in 1924 as “recurrent and progressive dermatofibromas or fibrosarcomas of the skin” and was later characterized by Hoffman in 1925 as DFSP [4,5]. DFSP accounts for less than 0.1% of all malignancies and 1-6% of soft tissue sarcomas [6,7]. Previous studies differ on whether men or women are more commonly affected [1,8]. This tumor is most typically found in young and middle-aged patients; however, cases have been reported in all ages, including infants and elderly patients [1]. Almost half of the reported cases occur on the trunk, 30-40% of cases occur on proximal extremities, and 10-15% of cases occur on the head and neck [9,10]. DFSP involving the breast is extremely rare and breast imaging findings may mimic those of primary breast neoplasms [2,3].

Clinically, DFSP often presents as a small, firm, painless, flesh-colored, or erythematous plaque that slowly grows over time to eventually form single or multiple raised and coalescing nodules. These may remain confined to the skin or they may invade the subcutaneous tissue and underlying musculature [8]. Because it tends to be painless in its earlier stages, DFSP is frequently ignored by patients for months or even years, and previous studies indicate that the time from symptom onset to diagnosis may span decades [11]. In our patient’s case, she reported having an enlarging skin lesion for years and only sought care after it became painful and began to ulcerate.

Imaging findings on mammography and ultrasound can be non-specific and may resemble benign breast neoplasms such as fibroadenoma, particularly in cases where the tumor does not clearly originate in the dermis [2,3,11,12]. On mammography, DFSP typically presents as an exophytic, gently lobulated, and non-calcified mass with circumscribed margins. On ultrasound, the tumor tends to be hypoechoic relative to the surrounding fat, with intervening echogenic bands, an echogenicity pattern that reflects densely cellular nests of malignant spindle cells (low echogenicity) with tumor cells and surrounding fibrous tissue invading into subcutaneous fat (high echogenicity). Posterior acoustic enhancement is common due to the dense cellularity of the mass. Color Doppler evaluation often reveals intralesional hypervascularity, typically more peripheral than central [13]. Although our patient did not undergo further imaging studies to evaluate her breast lesion, previous studies have described the appearance of DFSP in the breast on other imaging modalities such as magnetic resonance imaging (MRI) and positron emission tomography-CT (PET-CT). On MRI, it tends to be a circumscribed, oval mass with a wide base originating in the skin, hypo- to isointense on T1, hyperintense on T2, and rapidly enhancing with contrast administration [2,14]. PET-CT findings, which have only been described in a few case reports, typically demonstrate mild-moderately increased uptake of FDG around DFSP lesions of the breast [2,14,15].

Definitive diagnosis of DFSP is accomplished with a punch biopsy or excisional biopsy. Histologic examination usually reveals bland spindle cells arranged in a storiform or intersecting pattern surrounded by a collagenous stroma, with rare mitotic figures of not more than 10 mitoses per 10 high-power fields. Spindle cells may be observed infiltrating into subcutaneous tissues and underlying muscle in a classic “honeycomb” appearance. Immunohistochemical staining of DFSP demonstrates positive CD34 expression, in most cases [16].

Treatment of DFSP is surgical resection with wide margins of 2-3 cm [17,18]. Local radiation or targeted chemotherapy with the PDGFR inhibitor Imatinib may be useful when surgical resection is not possible due to location or due to the patient's refusal of surgery. Localized radiation therapy may also be used in cases of incomplete surgical resection. Localized recurrence of DFSP is common, with recurrence rates ranging from 10-60%; the risk of recurrence is elevated in cases where surgical resection margins are inadequate. This is primarily due to the microscopic finger-like, invasive protrusions that are often observed in DFSP, which may not be appreciated grossly during surgical resection [19]. Furthermore, since surgery and scar formation are risk factors for the development of DFSP, these may contribute to the increased risk of recurrence in patients with a prior history of the disease [20]. Metastatic disease is rare, with local metastases observed in 6% of cases and distant metastases in only 1% of cases. Distant metastasis usually occurs only after multiple instances of local recurrence and spread. The widespread metastatic disease most often occurs in the lung, brain, bone, viscera, and soft tissues through the hematogenous spread, with regional lymph nodes seldom involved. Overall, DFSP has an excellent prognosis and high 10-year survival rates (>99%) if adequately and promptly excised [1,16].

## Conclusions

DFSP of the breast is an exceedingly rare condition that may mimic primary breast lesions on imaging. Within this case report, we have presented and discussed key imaging findings that are typically observed in DFSP within the breast. Although this tumor is typically slow-growing with a low incidence of metastatic disease, it has a high local recurrence rate and aggressive local resection is necessary to minimize the chance of recurrence.
